# Preliminary Experience with Small Animal SPECT Imaging on Clinical Gamma Cameras

**DOI:** 10.1155/2014/369509

**Published:** 2014-05-14

**Authors:** P. Aguiar, J. Silva-Rodríguez, M. Herranz, A. Ruibal

**Affiliations:** ^1^Molecular Imaging and Oncology Group, IDIS Research Institute, Travesía da Choupana s/n, 15706 Santiago de Compostela, Spain; ^2^Nuclear Medicine Department, University Hospital of Santiago de Compostela (CHUS), Travesía da Choupana s/n, 15706 Santiago de Compostela, Spain; ^3^Molecular Imaging Group, Faculty of Medicine, University of Santiago de Compostela (USC), 15782 Santiago de Compostela, Spain; ^4^Fundacion Tejerina, Calle de José Abascal 40, 28003 Madrid, Spain

## Abstract

The traditional lack of techniques suitable for *in vivo* imaging has induced a great interest in molecular imaging for preclinical research. Nevertheless, its use spreads slowly due to the difficulties in justifying the high cost of the current dedicated preclinical scanners. An alternative for lowering the costs is to repurpose old clinical gamma cameras to be used for preclinical imaging. In this paper we assess the performance of a portable device, that is, working coupled to a single-head clinical gamma camera, and we present our preliminary experience in several small animal applications. Our findings, based on phantom experiments and animal studies, provided an image quality, in terms of contrast-noise trade-off, comparable to dedicated preclinical pinhole-based scanners. We feel that our portable device offers an opportunity for recycling the widespread availability of clinical gamma cameras in nuclear medicine departments to be used in small animal SPECT imaging and we hope that it can contribute to spreading the use of preclinical imaging within institutions on tight budgets.

## 1. Introduction

The traditional lack of techniques suitable for* in vivo* imaging has induced a great interest in molecular imaging for preclinical research. Single photon emission computed tomography (SPECT) and positron emission tomography (PET) are the most commonly employed molecular imaging techniques. They are noninvasive imaging techniques used for* in vivo* measurement of biological tracers for the diagnosis and treatment of many diseases. The application of SPECT and PET imaging techniques in preclinical research has similar benefits as in clinical routine, being used in drug discovery and development, to assess drugs' effects prior to clinical trials and to provide a better understanding of animal models with prospective clinical applications [1, 2].* In vivo* imaging not only complements* ex vivo* techniques, but also proves advantageous in certain situations, for example, allowing more than one-time exams or repeated observations over extended periods of time. Furthermore and importantly, it reduces the number of animals used in preclinical research significantly. At present, existing preclinical imaging systems are able to provide high quality and very detailed images of small animals and are thus a natural complement to classical techniques such as autoradiography and microscopy. Nevertheless, the great interest of preclinical imaging has been hindered by the high cost of preclinical scanners, similar to their clinical counterparts, which is due to the major improvements in detector instrumentation and tomographic reconstruction required to image small animals.

In particular, molecular SPECT imaging is based on gamma-emitting radionuclides that are injected into the patient and then emit a single photon that is detected by using a collimator and a gamma camera. The collimator provides information on the direction where the disintegration occurred. The collimator used for preclinical SPECT imaging is a pinhole collimator, that is, with just one small hole, which creates magnified projections and therefore interesting effects for imaging small organs and small animals [3]. The magnification factor is defined as the ratio between the distance from the pinhole to the detector (focal distance) and the distance from the pinhole to the object (radius of rotation). Therefore, high magnification factors require low radius of rotation or high focal distances. The radius can be decreased at the expense of the size of the FOV. The focal distance can be increased by positioning the detector farther from the collimator, but this requires using larger detectors. Yet, as large detectors are pricy, the current trend in preclinical SPECT is to design low-magnification systems with very small pinhole diameters, in order to improve the spatial resolution despite decreasing the sensitivity [4–7]. Advanced preclinical SPECT scanners circumvent the low-sensitivity problem including multipinhole collimators and several gamma camera heads [8–11].

An option to decrease the cost of a preclinical SPECT scanner is to repurpose a clinical gamma camera. The detectors integrated in clinical gamma cameras are large and therefore high magnification factors can be used to improve the spatial resolution [12]. The problem is that they were designed to rotate around human patients, and, in preclinical imaging the animals to be scanned are typically ten times smaller. The rotating system of the clinical gamma camera should be extremely precise, which greatly increases the cost of the system. A low-cost solution is to keep static the gamma camera and rotate the animal [13, 14]. Following this trend, we have designed a portable device that can be attached to a clinical gamma camera to generate preclinical SPECT images.

In this paper, we present our preliminary experience with a portable device working coupled to a single-head clinical gamma camera and we assess its performance to be used for small animal SPECT imaging.

## 2. Materials and Methods

Firstly, we describe the design and structure of the small animal SPECT system, as well as the acquisition and reconstruction software. In the second part, we present an evaluation of the image quality based on phantom measurements and different* in vivo* animal studies.

### 2.1. Pinhole SPECT System: Design, Acquisition, and Reconstruction

**Figure 1:**
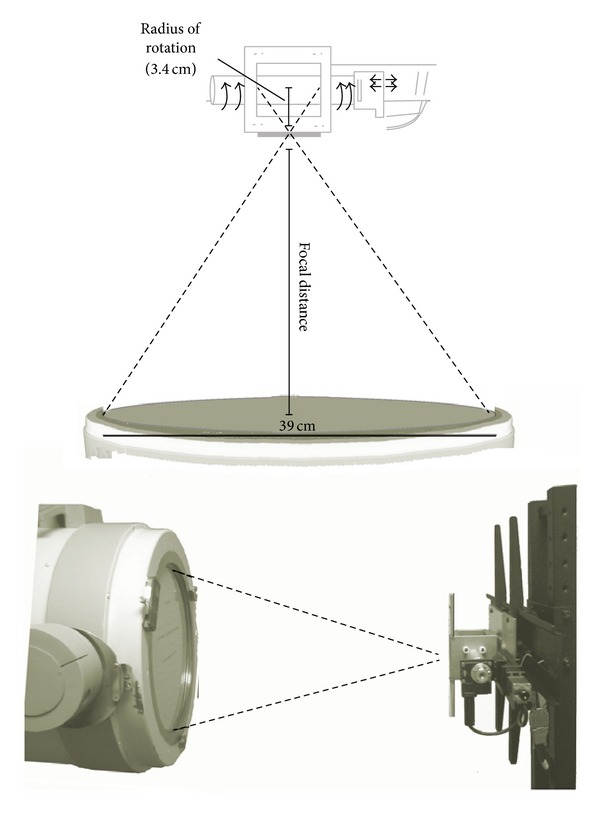
Scheme of a large detector with the portable device (top) and photograph of the clinical gamma camera working with the device, with the positioning system, animal holder, and the collimator.

The pinhole SPECT system consists of a clinical gamma camera and a portable device (Figure 1). The clinical gamma camera is a single-head clinical Siemens Orbiter gamma camera (Siemens Medical Solutions, Inc., USA), available at our nuclear medicine department. The portable device is composed of the animal holder, the positioning system, and the pinhole collimator. The animal holder is a methacrylate plastic cylinder of 3.2 cm in diameter in order to support the animal being scanned. It is open to allow for easy access to the animal for anaesthetization and monitoring of vital constants. The positioning system consists of automated rotatory and linear stages. The collimator is a single pinhole 2 mm in diameter made of tungsten sheets. The entire device is mounted on a portable support that ensures that these components are kept in the selected positions with respect to each other. The device has to be placed in front of the gamma camera, and this portable support allows for relative movement between them, which in turn allows variations of the focal distance.

Firstly, the number of projections and the total duration of the study have to be selected. Then, data is acquired for the first projection and, when finished, the rotatory system moves the object to the next projection angle. Any number of projections (1–360) and any study duration time can be selected. Data is acquired in list-mode format, so corrections, such as energy and spatial linearity, and uniformity corrections can be performed. All experiments were carried out using a fixed radius of rotation of 3.4 cm, 60 projections (6° angular steps and 60 seconds per projection), an energy window of 110–170 keV, and bin size of 3.2 mm.

Projection data are then reconstructed using an iterative algorithm based on the ordered subsets expectation maximization (OSEM) algorithm. This reconstruction algorithm was developed by [7], validated by [15], and finally adapted to our system. It includes the effect of the pinhole aperture, septal penetration, an experimental model for intrinsic detector response, and a calibration of the geometric parameters that describe the acquisition. The reconstruction was performed with 100 × 100 × 50 voxels (0.32 × 0.32 × 0.64 mm^3^ voxel size), using 20 subsets and different numbers of iterations, ranging from 1 to 500; no postreconstruction filters were applied.

### 2.2. Phantom Experiments

Phantom experiments were performed to evaluate the system performance in order to define protocols for animal studies. It is well known that an optimal trade-off between contrast and noise must be found by choosing an optimal iteration number. Nevertheless, the appropriate number of iterations is dependent on several factors, such as the lesion size and the scan statistics. In theory each patient and each lesion may need an individual assessment of the appropriate number of iterations.

We have carried out a performance evaluation in terms of image noise, contrast, and number of iterations by using a cylindrical phantom of diameter 16 mm and length 3 cm that contains 6 rod inserts with diameters of 0.5, 1, 2, 3, 4, and 5 mm. The rods were filled with a solution of ^99m^Tc and water (activity concentration of 247 MBq/mL) and no activity in the background was added. Every section of the phantom was uniformly filled with the same activity concentration.

#### 2.2.1. Contrast

It was obtained by measuring the average counts within a cylindrical volume of interest centered in each rod and the average counts within a cylindrical volume of interest centred in the background. It represents a measure of the convergence of the reconstruction algorithm and depends on the size of the source of activity and the number of iterations.

#### 2.2.2. Image Noise

It was evaluated through the coefficient of variation, also called normalized image noise. It is defined as the ratio of the standard deviation to the mean value of the counts measured in a volume of interest. It was calculated for different iterations as the ratio of the average standard deviation to the mean counts in a cylindrical volume of interest of diameter 12 mm and height 5 mm centered in the uniform section of the phantom.

### 2.3. *In Vivo* Animal Studies

The results derived from phantom experiments in the previous section were used to find optimal protocols for animal studies. Two* in vivo* mouse studies were carried out in order to evaluate the performance of the portable device in small animal imaging.

#### 2.3.1. Whole-Body Bone Scan

The second study was a bone scan obtained by using a healthy laboratory mouse, scanned 2 hours after injection of 100 MBq of ^99m^Tc-HDP (hydroxymethane diphosphonate) in the tail vein. The whole-body scan was obtained by acquiring four 1 h bed positions, with decay correction applied by adequately increasing the projection time for each bed position. HDP uptake occurs as a function of skeletal blood flow and osteogenic activity and therefore HDP-SPECT can be used for imaging of areas of abnormal osteogenesis associated with malignant bone lesions. It is clear that the visualization of the mouse skeleton and the localization of small bone lesions require a better spatial resolution than the renal scan, and therefore a different protocol in terms of number of iterations should be used.

#### 2.3.2. Thyroid Scan

The third study was a thyroid scan. To perform this study, a healthy mouse was scanned 10 minutes after peritoneal injection of 37 MBq of ^99m^Tc. A single 1 h bed position was acquired over the intended region. The normal size of the mouse thyroid gland is about 2–4 mm and the dimension of each lobule can be less than 1 mm. The protocol for these studies should include a larger number of iterations in order to resolve submilimetric structures with high contrast.

## 3. Results

### 3.1. Phantom Experiments

**Figure 2:**
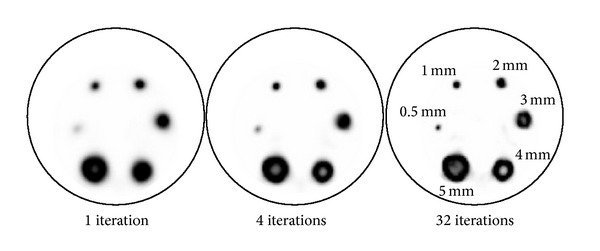
Hot rod phantom, reconstructed transaxial slices using 1, 4, and 32 iterations.

Figure 2 shows transaxial images of the hot phantom acquisition reconstructed using 1, 4, and 32 iterations. Of the six hot rods, the smallest 0.5 mm rod cannot be clearly identified after 1 iteration, but it becomes visible using larger number of iterations. The remaining rods are clearly identified after just 1 iteration, although 1 mm rod is rather blurred. Ring-type artefacts and overshoot are visible in the three largest hot rods (diameters 5, 4, and 3 mm) after 4 iterations and become more evident at larger number of iterations. These types of artifacts are likely caused by the Gibbs phenomenon.

**Figure 3:**
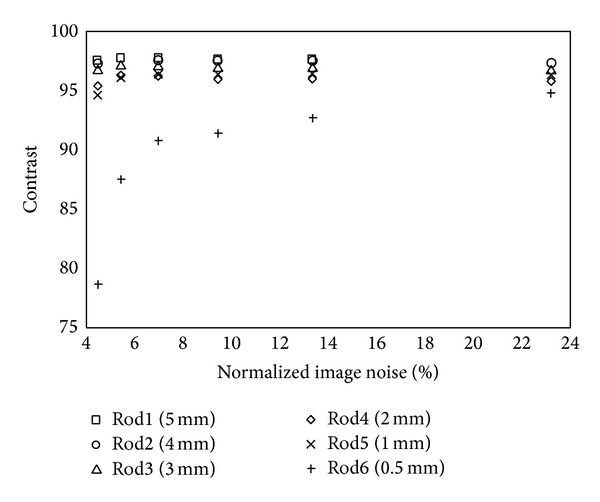
Hot phantom, contrast values measured for the different rod inserts at different numbers of iterations (1–32).

Figure 3 shows the contrast obtained for the different rod diameters as a function of the normalized image noise of the reconstructed image. As expected, the undesired effect of convergence is that the image noise values increase monotonically with the number of iterations; that is, the improvements in contrast are achieved at the expense of increased image noise. The contrast of the larger rod diameters (5 mm, 4 mm, 3 mm, and 2 mm) is already close to its maximum value (>95%) after iteration 1 and therefore at image noise values as low as 5%. Nevertheless, the convergence of the smaller rod diameters (1 mm and 0.5 mm) requires more iterations. The 1 mm rod was obtained with contrast values higher than 95% after iteration 4 and therefore image noise values of 7%. The 0.5 mm rod was obtained with contrast values higher than 95% after iteration 32 and therefore image noise values of 23%. In summary, our findings show that the number of iterations should be matched to diagnostic task of each study, taking into account considerations such as the required spatial resolution.

### 3.2. *In Vivo* Animal Tests

**Figure 4:**
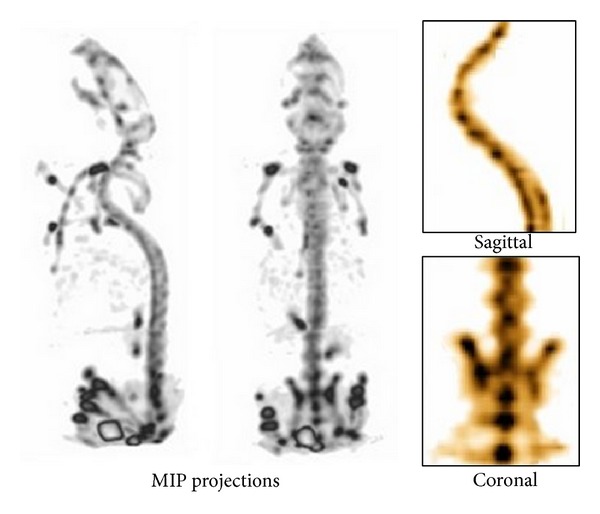
Healthy mice studies. HDP-SPECT: MIP projections (left); sagittal and coronal SPECT images (right), obtained 2 h after injection of ^99m^Tc-HDM.

Figure 4 shows the reconstructed images of ^99m^Tc-HDP mouse bone study, showing the maximum intensity projections (MIP) and sagittal and coronal views of the SPECT images. Detailed images of the bones were obtained, and even small structures such as the vertebrae were resolved by using a number of iterations equal to 4.

**Figure 5:**
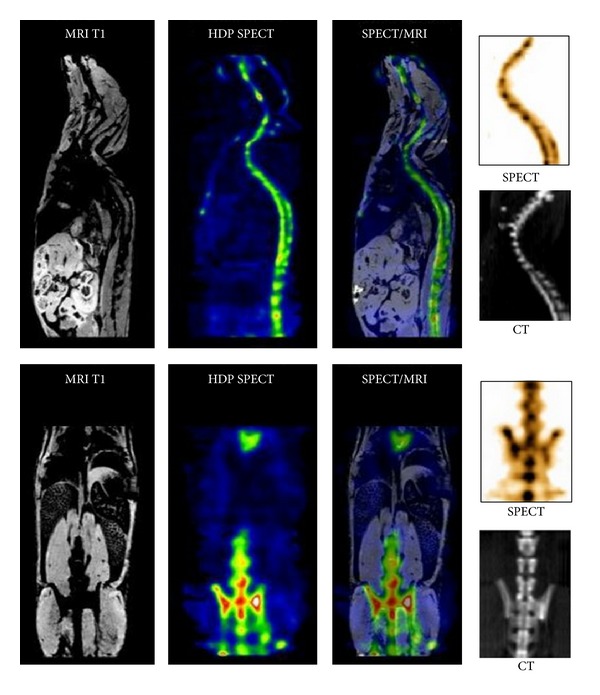
Healthy mice studies. SPECT/MRI and SPECT/CT.

Figure 5 shows multimodality images from the coregistration of the bone SPECT scan to MRI and CT images. The comparison with anatomical images (MRI and CT) shows that the functional information obtained with SPECT perfectly matches the bone structural information.

**Figure 6:**
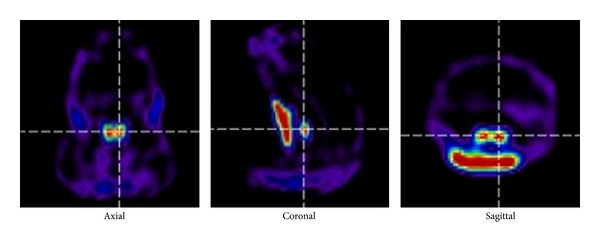
Healthy mice studies. Thyroid SPECT of sagittal, coronal, and axial images 10 min after injection of ^99m^Tc (right).

Figure 6 shows the reconstructed images from the ^99m^Tc mouse thyroid study. Clear images of the thyroid with high contrast were obtained, and the two thyroid lobes could be clearly resolved after 32 iterations.

## 4. Discussion

It is well known that the optimal contrast-noise trade-off has to be found by choosing an appropriate iteration number, which is dependent on factors such as the lesion size and the detected counts. The problem is that each patient and each lesion may need an individual assessment of the appropriate number of iterations. To overcome this, we have performed phantoms' studies to assess the quality of our images and the reconstruction procedure in different cases. Our findings showed that our device is capable of resolving with high contrast (>94%) structures larger than 2 mm at noise levels of 5% (iteration 1), structures about 1 mm at noise levels of 7% (iteration 4), and very small structures about 0.5 mm at noise levels of 23% (iteration 32). The latter results based on phantom experiments were used for defining optimal protocols in terms of number of iterations for* in vivo* animal scans. The whole-body bone scan was obtained after only 4 iterations and makes it possible to distinguish most of the bony structures appearing in the corresponding MRI and CT image. The thyroid scan allowed us to distinguish the two lobes of the mouse thyroid clearly separated. Unlike renal and bone scans, this study required 32 iterations in order to resolve the two thyroid lobes with high contrast. We should be aware that the normal size of each lobule of the mouse thyroid is about 1 mm.

We will discuss the strengths and weaknesses of our design. One of the positive features of our system is that it provides high contrast images of relatively small structures (about 1 mm) with an acceptable noise level (about 7%). This success comes from the fact that we have combined a large pinhole collimator (2 mm) with high magnification, in order to avoid degrading the spatial resolution. We feel that the outcome performance of our system is a great result as it is comparable to the trade-off between contrast and noise obtained from other pinhole-based prototypes [6, 13]. Nevertheless, the performance of our system is worse than the performance of the novel multipinhole-based commercial systems [16]. Due to this, animal tests reported here were obtained after administration of high doses and the total acquisition times about 1 h, which has to be mentioned as an important weakness of proposed preclinical imaging system compared to multipinhole-based commercial systems. Regarding this, our system could be used with larger pinhole diameters (3-4 mm), so that the sensitivity could be increased. Nevertheless, further studies should be carried out in order to analyze whether this gain in sensitivity can be sustained without a significant loss of spatial resolution. Among the main strengths of our device are also its versatility, portability, and simplicity. For example, the system is versatile to be used with a different gamma camera model and even with gamma cameras that are also being used in clinical practice. The simplicity of the design allows us to keep the cost to a small fraction of the tag price of a commercial system and it offers an opportunity for recycling the widespread availability of clinical gamma cameras in nuclear medicine departments to be used in small animal SPECT imaging. Among the weaknesses, it has to be also mentioned that some ring-type artifacts were observed, likely caused by the well-known Gibbs effect [17] and due to the truncation of a Fourier series of a discontinuous function during the reconstruction process. This was particularly relevant for larger rods, with diameters of 5, 4, and 3 mm, whereas it was not observed in rods with diameters of 2, 1, and 0.5 mm. This phenomenon has been extensively studied in a previous work [7], and the authors are investigating strategies to reduce its effects.

## 5. Conclusions

We have assessed the performance of a device that can be used with a clinical gamma camera to generate small animal SPECT images. Our findings, based on phantom experiments and animal studies, showed that the proposed system can be used for preclinical imaging with an image quality comparable to dedicated pinhole-based preclinical imaging scanners. The main strengths of our device are its versatility and portability, as it is not physically attached to the gamma camera and therefore can be used with a different gamma camera model and even with gamma cameras that are also being used in clinical practice. Furthermore, the simplicity of the design allows us to keep the cost to a small fraction of the tag price of a commercial system. For all these reasons and finally we feel that our system offers an opportunity for recycling the widespread availability of clinical gamma cameras in nuclear medicine departments to be used in small animal SPECT imaging and we hope that it can contribute to spreading the use of preclinical SPECT imaging within institutions on tight budgets.
